# Tailoring Whispering Gallery Lasing and Random Lasing in A Compound Cavity

**DOI:** 10.3390/polym12030656

**Published:** 2020-03-13

**Authors:** Zhiyang Xu, Junhua Tong, Xiaoyu Shi, Jinxiang Deng, Tianrui Zhai

**Affiliations:** College of Applied Sciences, Beijing University of Technology, Beijing 100124, China; xu.zhiyang@hotmail.com (Z.X.); jhtong@emails.bjut.edu.cn (J.T.); xyshi@bjut.edu.cn (X.S.)

**Keywords:** whispering gallery mode, random laser, polymer laser, compound cavity

## Abstract

A compound cavity was proposed to achieve both whispering gallery mode (WGM) lasing and random lasing. The WGM-random compound cavity consisted of a random structure with an annular boundary, which was fabricated by a method combining both inkjet printing and metal-assisted chemical etching methods. An ultrathin polymer membrane was attached to the WGM-random compound cavity, forming a polymer laser device. A transformation from WGM lasing to random lasing was observed under optical pumping conditions. The laser performance could be easily tailored by changing the parameter of the WGM-random compound cavity. These results provide a new avenue for the design of integrated light sources for sensing applications.

## 1. Introduction

Microcavity lasers have attracted significant attention due to their small size, low threshold, and broad universal applications, e.g., displays, imaging, sensing, and on-chip optical communication [1,2,3,4]. A large variety of microcavities have been designed to fabricate microscale lasers, e.g., Fabry-Perot, distributed feedback (DFB), and random and whispering gallery mode (WGM) lasers [5,6,7,8,9]. Each of them had distinctly different resonance mechanisms and structures. Among them, the random lasing was achieved by strong multiple scattering of light in disordered gain media. A particular advantage of the random laser was that they can be produced on small size, cavity-less, and low spatial coherence [10,11,12]. The WGM lasing was achieved via total internal reflection, which resulted in low threshold and narrow linewidth lasers [13,14,15]. Different kinds of laser were expected to be integrated in a compound cavity to explore the miniaturization and interactions of microcavity lasers. Recently, the integration of Fabry-Perot-WGM, Fabry-Perot-random, DFB-random, and WGM-random were reported, respectively [16,17,18,19]. Liu et al. reported a stable hybrid WGM/random lasing, which was achieved from a single microsized SiO_2_ sphere with all-inorganic perovskite CsPbBr_3_-SiO_2_ quantum dots (QDs) embedded [19]. However, the evolution of WGM laser to random laser in a WGM-random cavity has not been explored systematically. Furthermore, it remains a challenge to tailor both WGM and random lasers in one single device.

In this work, a WGM-random compound cavity was proposed to achieve WGM lasing and random lasing in a single laser device. The compound cavity consisted of a disc-shaped Si structure decorated with random shaped mesopores, which was fabricated by inkjet printing and metal-assisted chemical etching (MACE) method. For MACE of silicon, etching was confined to a small region surrounding metal catalyst templates [20]. Then a polymer membrane which acted as a gain medium was attached to the compound cavity to form the polymer laser device. Upon optical pumping, WGM lasing and random lasing were observed simultaneously. During the inkjet printing process, the diameter of the Si microdisc related to the concentration of photoresist droplets. The characteristic of the WGM lasing was modified by changing the diameter of the Si microdisc.

## 2. Structures and Methods

**Figure 1:**
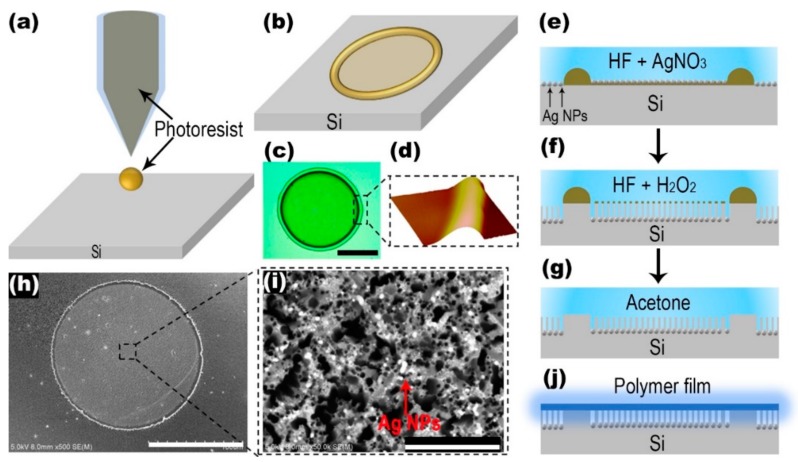
The fabrication process of the whispering gallery mode (WGM)-random compound laser by using inkjet printing and metal-assisted chemical etching (MACE) method. (**a**) Inkjet printing process of the diluted photoresist ink. (**b**) Schematic illustrating the ultrathin photoresist microdisc with a higher ring-shaped structure at the boundary. (**c**) Microscopic image of the photoresist microdisc. The scale bar is 60 μm. (**d**) The atomic force microscopy (AFM) image of the higher ring-shaped structure at the boundary of microdisc. (**e**) The process of depositing a layer of Ag nanoparticles on the surface. (**f**) The etching progress of the disc-shaped Si structure decorated with random-shaped mesopores. (**g**) The cleaning process of the photoresist. (**h**) The SEM image of the disc-shaped Si structure decorated with mesopores. The scale bar is 100 μm. (**i**) The SEM image of the enlarged view of the random shaped mesoporous structure. The scale bar is 1 μm. Ag nanoparticles (NPs) are indicated by the red arrow. (**j**) Attaching a light-emitting polymer film on the disc-shaped Si structure decorated with mesopores.

In the experiment, boron-doped p-type Si (100) wafers with resistivity (*ρ*)1-10 Ω·cm were used as a substrate. Figure 1 depicts the fabrication process of the WGM-random compound lasing device by using the inkjet printing and MACE method. The ink for inkjet printing was prepared by diluting photoresist with acetone. In order to fully dissolve the photoresist in acetone, the inks were ultrasonic oscillated for 10 min. The diameter of Si microdiscs can be controlled by the volume ratio of photoresist ink to acetone. A high-precision printer (Microfab JETLAB 4, Microfab Technologies Inc., Shanghai, China) equipped with a 60 μm diameter piezoelectric-driven inkjet nozzle was employed to print the photoresist droplets on the substrate, as shown in Figure 1a. Due to the coffee-ring effect [21,22], the printed photoresist droplet formed an ultrathin microdisc with higher ring-shaped structures at the boundary, as shown in Figure 1b. The microscopic (OLS4100, Olympus, Tokyo, Japan) image of the photoresist microdisc is shown in Figure 1c. The diameter of the microdisc was about 142 μm and the ratio of photoresist to acetone was 1:10. The atomic force microscopy (AFM, Alpha 300, WlTec, GmbH, Frankfurt, Germany) image in Figure 1d shows that the thickness of the higher ring-shaped structure at the boundary of the microdisc was about 1 μm, which could resist etching process of MACE. 

**Figure 2:**
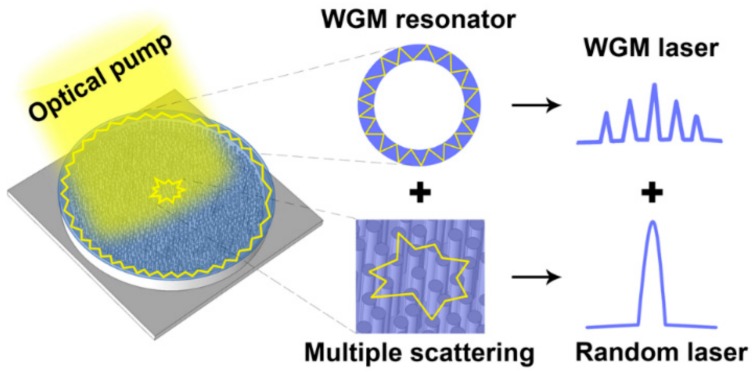
Schematic diagram of the WGM-random compound cavity.

A two-step MACE process was used, consisting of depositing Ag catalyst in the solution to AgNO_3_-HF and etching in the HF–H_2_O_2_ solution, as shown schematically in Figure 1e–g. The Si wafer covered with the micromask of photoresist was first immersed in a solution containing 4.8 M HF and 0.004 M AgNO_3_ for 1 min. During this process, a layer of Ag nanoparticles (Ag NPs) was deposited on the surface of the Si wafer except for the boundary of the photoresist microdisc, as shown in Figure 1e. The boundary of the photoresist microdisc was thick enough to resist the oxidation-reduction reaction between silicon and silver ions in the mixture of AgNO_3_ and HF. Subsequently, the Ag NPs-coated Si sample was etched in a solution containing 4.8 M HF and 0.04 M H_2_O_2_ for 8 min. With the progress of the etching, the Ag NPs gradually etched down, and the disc-shaped Si structure decorated with random shaped mesopores was formed, as illustrated in Figure 1f. To remove the circular photoresist dots on the Si surface, the Si wafer was cleaned by using acetone. Figure 1h,i show the SEM (Hitachi S-4800, Hitachi, Tokyo, Japan) images of the disc-shaped Si structure decorated with mesopores and the enlarged view of the randomly shaped mesoporous structure, respectively. The diameter of the Si disc was about 142 μm. There remained Ag NPs at the top of the mesoporous structure, which may have enhanced the scattering effect of random lasing. Finally, a layer of the light-emitting polymer membrane (15 mm × 15 mm) with a thickness of nearly 200 nm, poly [9,9-dioctylfluorenyl-2,7-diyl] (PFO, American Dye Source Inc., Baie DDUrfe, QC, Canada), was attached to the disc-shaped Si structure decorated with mesopores to form the WGM-random compound lasing device. The schematic of the whole system is demonstrated in Figure 2. The WGM and the random lasing mode were supported by the two resonators in the compound cavity, respectively. 

## 3. Results

**Figure 3:**
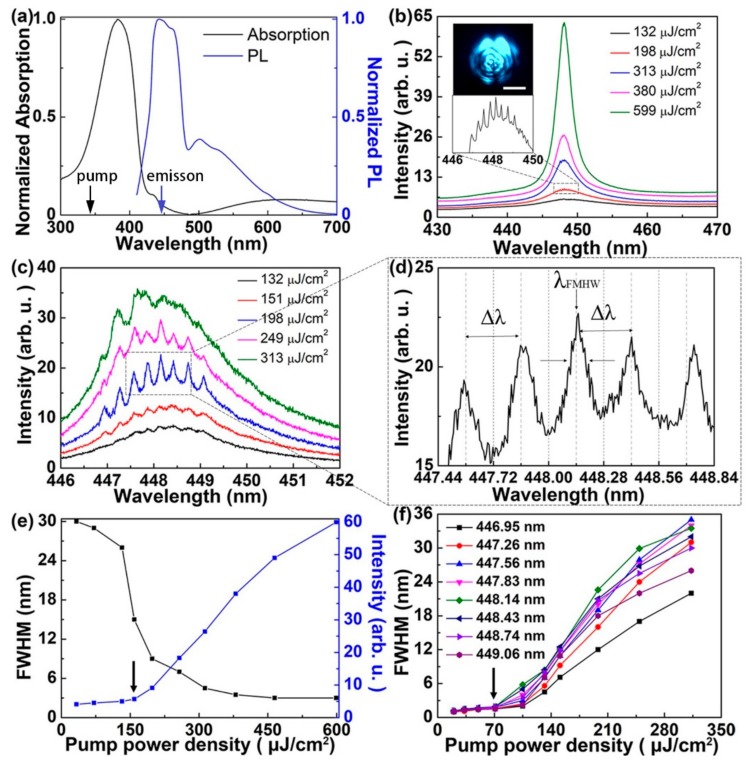
Spectral characteristics of the WGM-random compound laser. (**a**) Absorption and PL spectra of the poly [9,9-dioctylfluorenyl-2,7-diyl] (PFO) polymer film. The wavelength of the pump and lasing are indicated by the black and blue arrows, respectively. (**b**) Measured spectra of random lasing collected by the spectrometer at grating of 900 grooves/mm. The insets in denotes the enlarged view of the emission peak at the pumping energy of about 198 μJ/cm^2^ and the microscopic image of pumping laser spot. Scale bar: 100 μm. (**c**) Measured spectra of WGM lasing. (**d**) Enlarged view of WGM lasing modes. (**e**) Output intensity and linewidth of the random laser as a function of the pump energy. (**f**) Output intensity of the WGM lasing as a function of the pump energy. The thresholds of the WGM lasing and random lasing are indicated by the black arrows.

The absorption and photoluminescence (PL) spectra of PFO are plotted in Figure 3a. The broad absorption peak (red curve) was observed at 382 nm and the PL spectrum (blue curve) was centered at about 445 nm with the full width at half maximum (FWHM) of about 30 nm. There was little overlap between the absorption and PL spectra, which implied that the self-absorption of the PL emission was very weak. The pump wavelength was chosen within the absorption spectrum. The wavelengths of the pump and the emission are denoted by the black and the blue arrows, respectively, as shown in Figure 3a. During the spectral measurements, a short-pulsed diode-pumped solid-state laser (343 nm, 1 ns, 50 Hz, Coherent Inc., Santa Clara, CA, USA) was employed as a pump source. The pumping laser spot was focused by a 20× objective lens onto the top surface of the WGM-random compound lasing device. The photoemissions were collected and collimated by the same objective lens, and finally coupled to a spectrometer. As shown in the inset of Figure 3b, the beam diameter was about 0.2 mm, which was measured by a knife-edge method [23]. Note that the absorption of the Si structure affected the lasing properties. On one hand, it might decrease the emission intensity and increase the thresholds. On the other hand, it might help define the cavity by absorbing light outside the cavity. Such effect of absorption on defining the cavity has been discussed systematically [24], where very high concentration dye was used to confine the light in a small volume. Figure 3b shows the output intensity of the WGM-random lasing under different pulse pumping energy. The broad emission spectrum began to narrow when the pumping energy excessed the laser threshold. The emission peak could be observed at 448 nm and the FWHM of the emission peak (λ_FWHM_) was about 3 nm. Moreover, the inset in Figure 3b denotes the enlarged view of the emission peak at the pumping energy of about 198 μJ/cm^2^.

In order to investigate the lasing modes, a high spectral resolution spectrometer with 0.01 nm was used to record the lasing spectra from 446 to 452 nm. When the pump energy exceeded 70 μJ, several sharp peaks could be observed on the emission spectra and the output intensity increased significantly with increasing pump energy. Figure 3c shows the intensity of the sharp peaks with different pulse pumping energy collected by the spectrometer with high resolution. Several sharp peaks centered at 446.95, 447.26, 447.56, 447.83, 448.14, 448.42, 448.74 and 449.06 nm appeared over the spontaneous emission band. With the pumping energy increased, the intensity of spacing peaks increased and gradually reached saturation. The enlarged view of the spacing peaks at the pumping energy of about 198 μJ/cm^2^ is shown in Figure 3d. The characteristics of the spacing peaks were comparable with the reported polymer micro-ring lasers, suggesting the occurrence of WGM lasing action [25,26]. The spacing between two adjacent peaks ∆λ were almost the same, at about 0.28 nm. Spectral separation of laser lines of microresonators where light is confined in the form of WGM is expressed by ∆λ = λ^2^/(nπD), where λ is the emission wavelength, n is the effective refractive index of the cavity, and D is the diameter of microresonator. D could be estimated from the SEM image, which was about 142 μm. Then, n could be calculated as 1.61, which was approximate with the experimental value of PFO (1.64).

Figure 3e presents the plots of emission intensity and the FWHM as a function of pumping energy. For pump energy below 70 μJ, weak and extensive spontaneous emission from the WGM-random compound cavity could be observed. There was a nonlinear variation between the FWHM and pump energy, which is a signature for the occurrence of random lasing. The threshold for the random lasing was around 140 μJ/cm^2^ and the λ_FWHM_ rapidly narrowed to 3 from 30 nm over their threshold. The plots of WGM emissions intensity at 446.95, 447.26, 447.56, 447.83, 448.14, 448.42, 448.74 and 449.06 nm as a function of pumping energy are shown in Figure 3f. The threshold for the WGM lasing was around 70 μJ/cm^2^ and the λ_FWHM_ rapidly narrowed to 0.1 nm over their threshold.

The thresholds of WGM and random lasing in the compound microresonator were comparable. The threshold of the WGM lasing was near 70 μJ/cm^2^, while the threshold of the random lasing appeared to be 140 μJ/cm^2^. It could be attributed to the differences in photon confinement through comparison of cavity quality (Q) factors [27,28], where Q = λ/λ_FWHM_. For WGM lasing, photons propagate along the PFO microdisc via total internal reflection with the guided modes, of which the Q factor is measured to be higher than 3×10^3^. It is approximately an order of magnitude larger than the part of random lasing, in which the mesoporous structure increased the scattering of photons generated in the gain material. In other words, the main difference between the random and WGM laser was the Q factor. Since the Q factor of WGM cavity was much higher than that of the random cavity, the WGM laser had a lower threshold and a smaller λ_FWHM_ than the random laser.

**Figure 4:**
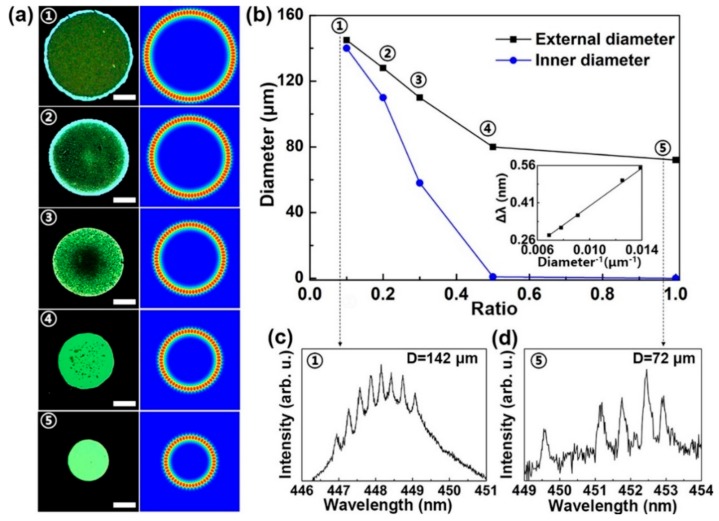
Tunability of the WGM-random compound laser. (**a**) Microscopic images of the Si microdiscs and the simulation of the field patterns of high-Q mode in discs ①–⑤. Scale bar: 30 μm. (**b**) The diameter of Si microdiscs as a function of the ratio of photoresist to acetone. The inset shows the dependence of mode spacing (∆λ) on the reciprocal of the microdisc diameter. Measured emission spectra of the microdiscs (**c**) ① and (**d**) ⑤.

The WGM-random compound laser was achieved by attaching a layer of light-emitting polymer membrane on the disc-shaped Si structure decorated with mesopores. In principle, the characteristics of WGM-random compound lasing is mainly determined by the sizes of bottom Si microdiscs and the mesoporous structure [29]. In this case, the mode spacing of WGM-random compound lasing could be controlled by fabricating Si microdiscs with different diameters. Figure 4a shows the microscopic image of the Si microdisc. The respective diameters of discs marked ①–⑤ were about 142, 128, 109, 80 and 72 μm, and the field patterns of high-Q mode in discs ①–⑤ were also simulated. Figure 4b shows the diameter of Si microdiscs as a function of the ratio of photoresist to acetone. The diameter of Si microdiscs was controlled by the diluting ratio of photoresist ink to acetone. Moreover, the laser emissions were confirmed from all the microdisc cavities. The lasing mode spacing increased from 0.28 to 0.31, 0.36, 0.5, and 0.55 nm. The dependence of mode spacing on the reciprocal of microdisc diameter is summarized in the inset in Figure 4b, indicating a linear relationship could be found as ∆λ = 40D^−1^. Note that there were no random-shaped mesopores decorated on Si microdiscs when the diluting ratio of photoresist ink to acetone was lower than 0.5. Figure 4c,d illustrate the laser spectra of microdiscs ① and ⑤, respectively. Due to the smooth surfaces, there was no broad random lasing emission but only WGM laser in microdisc ⑤. 

**Figure 5:**
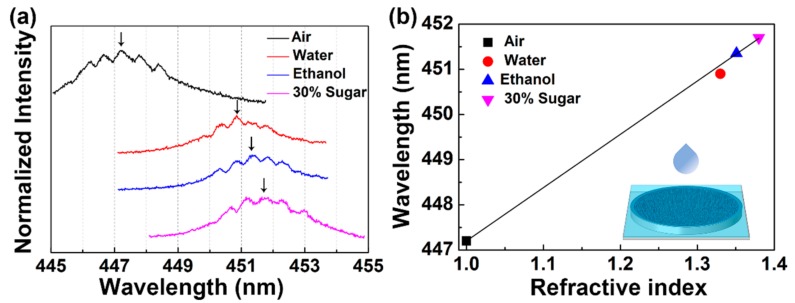
Sensing applications of the WGM-random laser. (**a**) Spectroscopic response of the WGM-random laser to the refractive index of the liquid. (**b**) Emission wavelength of the WGM-random laser as a function of the refractive index of the environmental medium. The inset showed the schematic of the laser sensor.

Microlaser-based sensors with high sensitivity and low detection limit indicate a high potential for practical applications. The proposed WGM-random laser can be used as a microlaser-based sensor, as shown in Figure 5. In the experiment, three different liquids, viz. deionized water (n = 1.33), ethanol (n = 1.36) and 30% sucrose (n = 1.38), were used to test the response of the laser device to the environmental medium. The diameter of the WGM-random compound cavity laser remained the same. The group of spacing peaks were shifted as a whole envelope with changing the ambient refractive index. So, the shift of the peaks could be recognized by the center wavelength of the envelope, as indicated by the black arrows in Figure 5a. The sensor signals in the WGM-random compound cavity laser were different from simple WGM-based sensors. With a narrowed photoluminescence peak under the spacing lasing peaks, the movement of the signal was more easily recognizable. 

As shown in Figure 5a, the emission wavelength of the WGM-random laser was tuned by changing the ambient materials from air to different liquids, where the emission peak redshift from 447.2 to 451.8 nm. Generally, the resonant wavelength of the WGM can be calculated from the following equation [30], λ_m_ = nπD/m, where m is the angular mode number, and D and n are the diameter and effective refractive index, respectively. In the case of constant D, a linear relationship between λ_m_ and n could be found from this equation. Figure 5b shows the spectral positions of the sensor signals due to the WGM-random laser as a function of the refractive index of the environmental medium of the miniaturized sensor device, where a linear relationship could be expressed as λ_m_ = 11.8n + 435.4. The schematic of the laser sensor is illustrated schematically in the inset of Figure 5b.

## 4. Conclusions

In conclusion, by combining the inkjet printing and MACE methods, a WGM-random compound cavity was fabricated to tailor the property of the WGM lasing and random lasing simultaneously. In the compound cavity, the Q factor of WGM lasing was an order of magnitude larger than the part of random lasing. The mode spacing of WGM-random compound lasing could be controlled by fabricating different diameter of Si microdiscs. Moreover, this WGM-random compound lasing device was employed to investigate the sensing properties of the external stimuli of liquids. These results could provide a new avenue for the design of WGM-random compound lasers with unique applications.
